# Effects of Two Types of 9-Month Adapted Physical Activity Program on Muscle Mass, Muscle Strength, and Balance in Moderate Sarcopenic Older Women

**DOI:** 10.1155/2018/5095673

**Published:** 2018-10-18

**Authors:** G. Piastra, L. Perasso, S. Lucarini, F. Monacelli, A. Bisio, V. Ferrando, M. Gallamini, E. Faelli, P. Ruggeri

**Affiliations:** ^1^Liguria Region Public Health Service-ASL4 Chiavarese, 16043 Chiavari, Italy; ^2^Department of Experimental Medicine (DIMES), University of Genoa, 16132 Genoa, Italy; ^3^Department of Internal Medicine and Medical Specialties (DIMI), University of Genoa, 16132 Genoa, Italy; ^4^Hospital Policlinic San Martino, Section of Geriatric Medicine, 16132 Genoa, Italy; ^5^Department of Neuroscience, Ophthalmology, Genetics, and Maternal-Infantile Sciences (DINOGMI), University of Genova, 16132 Genova, Italy; ^6^Ben-Essere Sport and Wellness Association Rapallo, Third-Sector Liguria Region Registry, 16132 Genoa, Italy

## Abstract

The present study aimed to evaluate the effects of two types of 9-month adapted physical activity (APA) program, based on a muscle reinforcement training and a postural training, respectively, on muscle mass, muscle strength, and static balance in moderate sarcopenic older women. The diagnosis of sarcopenia was done in accordance with measurable variables and cut-off points suggested by the European Working Group on Sarcopenia in Older People (EWGSOP). Seventy-two participants were randomly assigned to two groups: the muscle reinforcement training group (RESISTANCE) (n=35; 69.9 ± 2.7 years) and the postural training group (POSTURAL) (n=37; 70.0±2.8 years). Body composition, muscle mass, skeletal muscle mass index (SMI), and handgrip strength (HGS) were evaluated for sarcopenia assessment, whereas Sway Path, Sway Area, Stay Time, and Spatial Distance were evaluated for static balance assessment. Sixty-six participants completed the study (RESISTANCE group: n=33; POSTURAL group: n=33). Significant increases of muscle mass, SMI, and handgrip strength values were found in the RESISTANCE group, after muscle reinforcement program. No significant differences appeared in the POSTURAL group, after postural training. Furthermore, RESISTANCE group showed significant improvements in static balance parameters, whereas no significant differences appeared in the POSTURAL group. On the whole, the results of this study suggest that the APA program based on muscle reinforcement applied on moderate sarcopenic older women was able to significantly improve muscle mass and muscle strength, and it was also more effective than the applied postural protocol in determining positive effects on static balance.

## 1. Introduction

The older population in the world is predicted to increase by threefold within 50 years, from 600 million people in 2000 to over two billion in 2050. This increase may be viewed as one of society's greatest achievements, but preserving older adults' independence and quality of life remains one of the major clinical and public health challenges [1]. A severe change associated with aging is the progressive and apparently inevitable process of loss of muscle mass and strength [2] referred to as sarcopenia [3]. Although consensus definition and diagnosis criteria have not been reached, the bidimensional nature of sarcopenia is increasingly accepted, encompassing both the quantitative and qualitative declines of skeletal muscle and being characterized by a loss of muscle mass, strength, and power [4, 5]. This condition, which involves primarily women [6], has been associated with the atrophy of fast twitch type II muscle fibers and the substitution of functional tissues by adipose and fibrotic tissues that have reduced rates of protein synthesis, thus leading to reduced muscle efficiency [7].

The impaired state of health induced by sarcopenia is closely related to a risk of adverse outcomes such as physical disability, poor quality of life, impaired ability to perform activities of daily living [8, 9], and, mostly important, risk of falls and fractures, which represent the main causes of a downward spiral of loss of confidence and social withdrawal, which may ultimately lead to loss of independence [10]. The decrease in maximal muscle strength could be a main cause of postural instability [11], and the consequent reduced ability of old adults to adequately react to unexpected perturbations and to successfully regain balance is important intrinsic risk factor for falling [12–14].

Exercise has been shown to reduce the incidence of falls by 13% to 40%, which has led to a broad consensus among experts that older adults should be offered exercises that incorporate elements of balance and strength training [15]. Pieces of evidence support the notion that regular physical activity, in combination with appropriate nutritional support, is the most effective strategy for improving sarcopenia and physical function and preventing disability [16]. There are four types of exercises recommended in adapted physical activity (APA) for older adults: aerobic exercises, progressive resistance training, flexibility exercises, and balance training [17]. Particularly, progressive resistance training has been demonstrated to attenuate development of sarcopenia, by improving muscle size and function, reducing balance and flexibility problems, and reducing also the risk of development of other sarcopenia-related comorbidities [18]. The role of exercise in sarcopenia was investigated in several studies [19–22], but to the best of our knowledge, no standardized training protocol has been developed for healthy older people to induce positive quantitative and qualitative effects on muscle function and to improve balance. The aim of this study was to evaluate the effects on muscle mass and muscle strength and on static balance of two types of 9-month adapted physical activity (APA) programs based on muscle reinforcement training and postural training [23], respectively, in moderate sarcopenic older women.

## 2. Materials and Methods

### 2.1. Participants

Initially, 82 potential participants were assessed for eligibility from the community through advertisements in the notice board of the Public Health Service ASL 4 Chiavari, Italy, and in the local newspaper and the screening period was conducted between August and September 2016.

The recruitment process consisted of a medical evaluation to assess their good health and the absence of any contraindication to participation in adapted physical activity programs.

Participants were required to be at least 65 years of age and they were excluded if they had a pacemaker (due to the use of bioelectrical impedance analysis) or previous health problems such as neurological or cardiovascular diseases that would limit participation in the APA programs.

During enrolment, 10 potential participants were excluded, because 6 did not meet inclusion criteria and the other 4 declined to participate.

**Figure 1:**
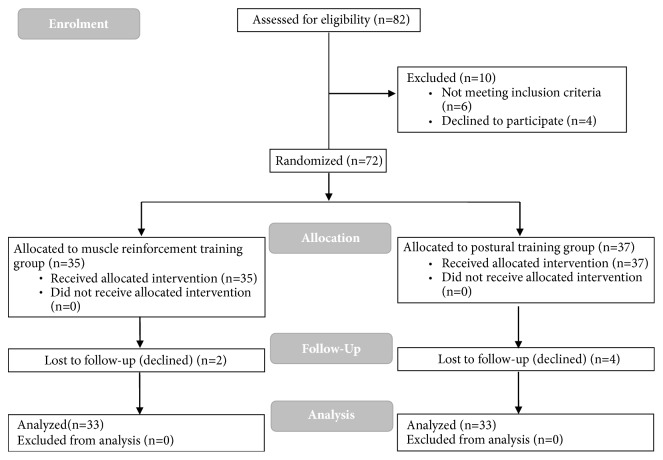
Flow diagram of the study.

At the end of the recruitment, 72 participants were enrolled for the study and they were randomly divided into two groups assigned to one of the two APA programs: muscle reinforcement training group (RESISTANCE) (n=35) and postural training group (POSTURAL) (n=37). Two out of 35 participants of the RESISTANCE group and 4 out of 37 participants of the POSTURAL group did not complete the training programs. A flow diagram of the study is reported in Figure 1.

The participant characteristics of the two groups at baseline are reported in Table 1.

The experimental protocol was approved by the Ethics Committee of the University of Genoa and, after explaining the aim and the procedure of the study, all participants gave their informed consent for intervention.

### 2.2. Sample Size

Estimation of sample size for this investigation was performed using handgrip strength as one of our primary outcome measures. Sample size was estimated combining the normative data and the genuine change in grip strength determined in previous works [24, 25]. These assumptions generated a desired sample size of at least 30 participants. However, we recruited 72 participants, 35 in the RESISTANCE group and 37 in the POSTURAL group, to allow for drop-out during the intervention period.

### 2.3. Testing Procedures

Testing was conducted before (T0) and after (T1) the APA intervention.

#### 2.3.1. Food Questionnaire

Before starting the APA program, all the participants were asked to answer to a food questionnaire in order to check a balanced daily intake of nutrients.

#### 2.3.2. Anthropometry and Body Composition

Body mass and height were measured to the nearest 0.1 kg and 0.5 cm, respectively, using a mechanical column scale and a stadiometer. Body composition evaluation was performed using a bioimpedance scale (InBody 320, GBC BioMed, NZ) at least 2 hours after the meal and participants were required to wear light clothing with bare feet. After ensuring proper feet placement and hold of hand electrodes, participants were instructed to stay relaxed during the measurements, maintaining a normal standing position, with arms and legs extended. The parameters used to measure the body composition were weight (Kg), body mass index (BMI), and muscle mass (Kg).

The skeletal muscle mass (SM) was calculated with the regression equation (1) as described by Janssen et al. [26]:

(1) SM kg=Ht2R×0.401+gender×3.825+age×−0.071+5.102

where Ht is height in centimeters;* R *is BIA resistance in ohms; for gender, men = 1 and women =0; and age is in years.

The same authors defined SM index (SMI) considering SMI as SM (Kg), obtained from the above-mentioned prediction equation, adjusted for the squared height (SM/height^2^, Kg/m^2^).

#### 2.3.3. Sarcopenia Assessment

Sarcopenia was diagnosed in accordance with EWGSOP criteria [8]. Particularly, we adopted the following measurable variables and cut-off points to diagnose sarcopenia presented in the above cited EWGSOP report: SMI, using BIA predicted skeletal muscle mass (SM) equation (SM/height^2^), and the handgrip strength. Concerning SMI, the cut-off values used by the EWGSOP were moderate sarcopenia when SMI is between 8.51 and 10.75 kg/m^2^ (men) or 5.76 and 6.75 kg/m^2^ (women) and severe sarcopenia when SMI is ≤8.50 kg/m^2^ (men) or ≤5.75 kg/m^2^ (women) [27]. HGS cut-off values for the diagnosis of sarcopenia were ≤ 30 kg (men) and ≤ 20 kg (women) [8, 27]. All the participants in our study showed a moderate sarcopenia. Muscle strength was assessed by handgrip strength, a proxy index of overall muscle strength [28], measured with a Jamar hydraulic hand-held dynamometer (Sammons Preston, Rolyan, Bolingbrook, IL, USA) with the second handle position for all participants and expressed in kg [29]. Additionally, the Jamar hand dynamometer has been shown to have acceptable concurrent validity in young and adults [30, 31]. Participants seated on a chair with their feet flat on the floor, holding the handgrip with their wrist in line with their elbow, and they were instructed to press the dynamometer as hard as possible. Participants performed a maximum voluntary isometric contraction of finger flexor muscles, three measurements were taken with 10-second intervals between each trial for both body sides (dominant and nondominant), and mean value of the two body sides was indicated as whole body handgrip strength (HGS). The maximum values were considered for statistical analysis [32].

#### 2.3.4. Static Balance

All participants performed the balance assessment on a static force platform (ARGO, RGM Medical Devices S.p.A., Genoa, Italy). The ARGO static force platform has a large platform surface area (600 x 600 mm) and a high sampling frequency (100 Hz). This platform, even with short measurement times, allows a reliable harmonic analysis of sway density parameters [33].

Participants were asked to stand with their feet joined and parallel on the barefoot, with their arms hanging loose at sides, and with closed mouth and unclenched teeth, looking at a target placed at their eye level about 1 m in front of them, in an acoustically isolated room and darkened when they were in closed eyes (CE) condition. Participants executed two trials in two different randomized modalities: with open eyes (OE) and with closed eyes (CE). Each assessment lasted 40 seconds preceded by a 5-second waiting time that allowed the participants to become familiar with the position, thus reducing the adaptation artifact [34, 35]. The parameters used to evaluate the static balance were as follows:

(1) Sway Path (SP) (mm/s), defined as Length of the Sway Path, normalized with respect to the duration of the acquisition interval;

(2) Sway Area (SA) (mm^2^/s), defined as the Sway Area by the radius connecting each subsequent point of the statokinesigram to the average position of the Centre of Pressure (COP), normalized with respect to the duration of the acquisition interval;

(3) Stay Time (ST) (s), the mean Stay Time spent by the COP trace in the neighbourhood of each peak, over the observed sway of each subject;

(4) Spatial Distance (SD) (mm), the average displacement of the COP trace between one peak and the next one.

### 2.4. APA Exercises

The two different APA programs were performed twice a week, for 36 weeks, and every session lasted for 60 minutes. The APA sessions were performed in a gym and conducted by an instructor in groups of a maximum of 20 participants.

Each session of the muscle reinforcement training was divided into three phases: (1) standing 15′ warming up and motor coordination exercises; (2) standing/on the ground 30′ muscle toning at low/moderate intensity for different muscular districts (primarily abdominal and both lower and upper limbs) with low weight loads (0.5, 1, or 1.5Kg); (3) cooling down 15′ and relaxation/stretching of the muscle systems with specific exercises [23].

Each session of the postural training was divided into three phases: (1) standing 10′-15′ cardiovascular activation, shoulder and coxofemoral joints mobilization, lower limbs reinforcement; (2) sitting 10′-15′ neck and shoulders mobilization; (3) on the ground, 5-30′ spine mobilization, abdominal muscles reinforcement, gluteal and spine extensor muscles reinforcement, spine stretching, hamstring and psoas muscle reinforcement and self-stretching, and final relaxation [23].

Both muscle reinforcement exercises and postural exercises were adapted to the participant's abilities and were progressive in repetitions and difficulties over time.

### 2.5. Data Analysis

Shapiro-Wilcoxon tests were used to evaluate whether the outcome variables were normally distributed.

SM, SMI, and HGS of the two groups were normally distributed and were compared before (T0) and after (T1) the intervention by means of a repeated measure ANOVA, with TIME (2 levels, T0 and T1), as within-subject factor, and GROUP (2 levels, RESISTANCE and POSTURAL), as between-subject factor. Newman-Keuls post hoc test was used to evaluate significant interactions.

Static balance data of the RESISTANCE and POSTURAL groups acquired with CE and OE were not normally distributed. Therefore, Mann-Whitney tests were used to evaluate differences between groups at each time epoch (T0 and T1), whilst Wilcoxon tests were applied to assess changes in each group. Significance for all procedures was set at a level of 0.05.

Data are presented as mean ± SD for normally distributed data and as median associated with the interquartile range for not normally distributed data.

## 3. Results

### 3.1. Effects on Sarcopenia

The statistical analysis on lean mass expressed as % of the total weight and in kg showed a significant effect of the factor TIME (%:* F(1,64)=22.70, p<0.001*; kg:* F(1,64)=18.25, p<0.0001*). Furthermore, a significant TIME*∗*GROUP interaction appeared (%:* F(1,64)=24.82, p<0.0001*; kg:* F(1,64)=29.53, p<0.0001*), and post hoc comparisons revealed a significant increase of lean mass values from T0 to T1 only in the RESISTANCE group (mean±SD; %: T0=30.10±8.44 %, T1=33.11±7.29 %; kg: T0=19.50±6.59 kg, T1=21.25±6.05 kg), whilst not significant differences appeared in the POSTURAL group (mean±SD; %: T0=30.52±5.93 %, T1=30.45±5.51 %; kg: T0=19.85±7.39 kg, T1=19.63±6.49 kg).

**Figure 2:**
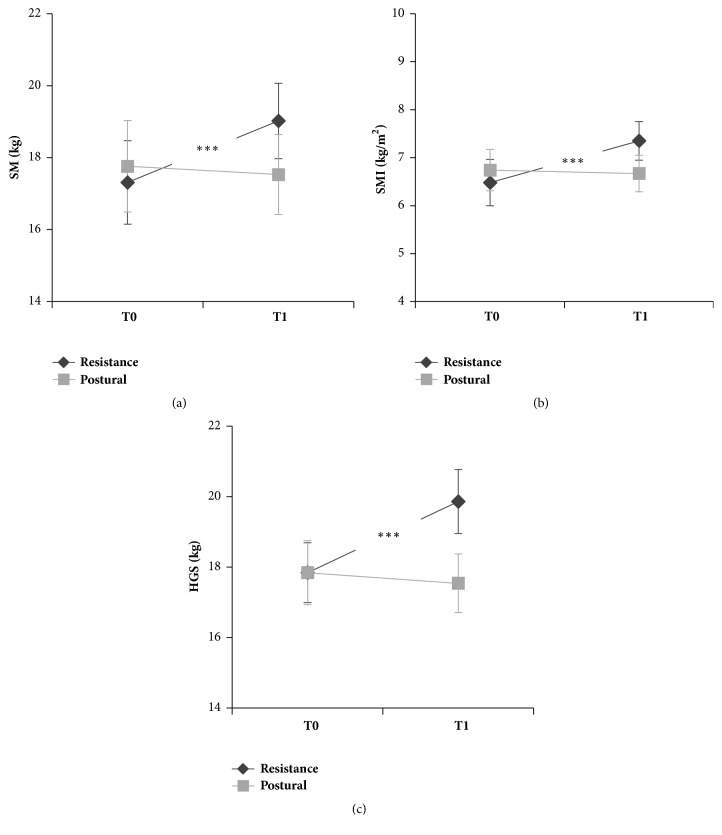
Values of skeletal muscle mass (SM) (a), skeletal muscle mass index (SMI) (b), and handgrip strength (HGS) (c) of the muscle reinforcement training group (RESISTANCE, black lines) and postural training group (POSTURAL, grey lines) before (T0) and after (T1) the intervention. Values are means ± SE. *∗∗∗* indicates p<0.001.

Data concerning skeletal muscle mass (SM) values before (T0) and after (T1) the intervention program are represented in Figure 2(a). The results of the statistical analysis showed a significant main effect of the factor TIME (*F(1,64)=17.76, p<0.001*) and a significant TIME*∗*GROUP interaction (*F(1,64)=29.04, p<0.0001*). Post hoc test revealed a significant increase of SM value only in the RESISTANCE group (mean±SD: T0=17.31±1.16 kg, T1=19.02±6.58 kg,* p<0.001*), whilst no differences were found between T0 and T1 in the POSTURAL group (mean±SD: T0=17.59±7.31 kg, T1=17.53±6.39 kg).

The statistical analysis on SMI values showed a significant effect of the factor TIME (*F(1,64)=19.89, p<0.0001*) and a significant TIME*∗*GROUP interaction (*F(1,64)=29.20, p<0.0001*). A significant increase of SMI values was found only after muscle reinforcement program (mean±SD: T0=6.48±2.75 kg/m^2^, T1=7.36±2.31 kg/m^2^,* p<0.001*). No significant differences appeared after POSTURAL training (mean±SD: T0=6.74±2.46 kg/m^2^, T1=6.67±2.17 kg/m^2^) (see Figure 2(b)).

Results of the handgrip test are represented in Figure 2(c). The statistical analysis on the mean of HGS of both hands showed a significant effect of the factor TIME (*F(1,64)=7.94, p<0.01*) and a significant interaction between TIME and GROUP (*F(1,64)=14.37, p<0.001*). A significant increase of HGS values was found only in the RESISTANCE group (mean±SD: T0=17.84±4.91 kg, T1=19.86±5.22 kg,* p<0.001*) and no differences appeared in the POSTURAL group (mean±SD: T0=17.84±5.25 kg, T1=17.55±4.85 kg).

### 3.2. Effects on Balance Parameters

**Figure 3:**
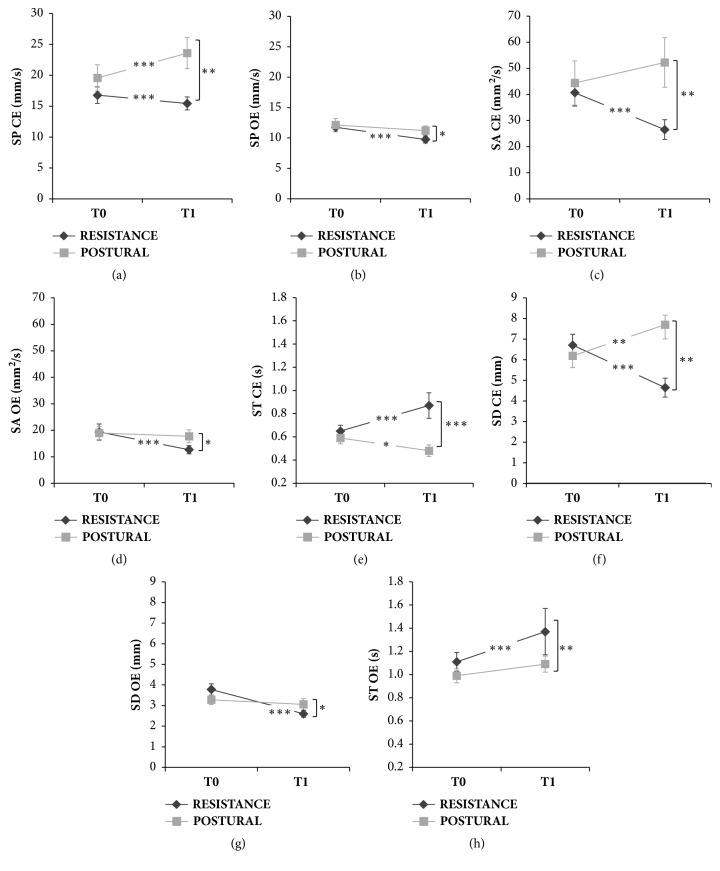
Static balance data of the RESISTANCE and POSTURAL groups, acquired with closed eyes (CE) and open eyes (OE) before (T0) and after (T1) the intervention. SP: Sway Path; SA: Sway Area; ST: Stay Time, and SD: Spatial Distance. Values are medians ± SE. *∗∗∗* indicates p<0.001, *∗∗* indicates p<0.01, and *∗* indicates p<0.05.

The static balance data are represented in Figure 3 and the descriptive statistics are reported in Table 2. The results of the Mann-Whitney test comparing the parameters of the two groups showed significant differences after the treatment, whilst no differences were observed at baseline. Furthermore, the results of the Wilcoxon test comparing the parameters related to each group acquired in T0 and T1 epochs revealed significant differences between the effects evoked in the two groups. Hereafter we will report only significant results.

#### 3.2.1. Sway Path (SP)

Wilcoxon tests comparing the Sway Path acquired with close eyes (SP CE) before and after treatments revealed a significant decrease of its value after muscle reinforcement training (*Z=3.53, p<0.001*) and a significant increase after the postural training (*Z=3.29, p<0.001*). When the test was repeated with eyes open a significant decrease was observed after muscle reinforcement training (*Z=3.94, p<0.001*), whilst no differences were observed in the POSTURAL group. The different effects of the two treatments appeared also when comparing Sway Path values at T1 in both conditions (CE and OE); indeed, SP in RESISTANCE group was significantly lower than SP in POSTURAL group (CE:* Z=-3.23, p<0.01*; OE:* Z=-2.46, p<0.05*). Data are represented in Figures 3(a) and 3(b).

#### 3.2.2. Sway Area (SA)

In the RESISTANCE group Sway Area decreased significantly after the treatment in both conditions (CE:* Z=5.01, p<0.001*; OE:* Z=4.89, p<0.001*), whilst no differences were observed in the POSTURAL group. Furthermore, the Mann-Whitney test comparing SA at T1 showed in both conditions that SA associated with the RESISTANCE group was significantly lower than that associated with the POSTURAL group (CE:* Z=-2.96, p<0.01*; OE:* Z=-2.23, p<0.05*). Data are represented in Figures 3(c) and 3(d).

#### 3.2.3. Stay Time (ST)

After the intervention, ST increased significantly only in the RESISTANCE group in both conditions (CE:* Z=4.01, p<0.001*; OE:* Z=4.69, p<0.001*), whilst a significant decrease after the postural training was observed only in CE condition (*Z=2.49, p<0.05*). The comparison between the two groups at T1 showed that ST associated with RESISTANCE group was higher than that in the POSTURAL group in both conditions (CE:* Z=3.65, p<0.001*; OE:* Z=2.64, p<0.01*). Data are represented in Figures 3(e) and 3(f).

#### 3.2.4. Spatial Distance (SD)

In the RESISTANCE group the results of the Wilcoxon tests showed a significant decrease of SD value in both conditions (CE:* Z=4.44, p<0.001*; OE:* Z=4.74, p<0.001*), whilst a significant increase was obtained after the postural training only in CE condition (*Z=2.72, p<0.01*). Data are represented in Figures 3(g) and 3(h).

## 4. Discussion

The results of this study, performed in moderate sarcopenic older women, demonstrate that the RESISTANCE group showed significant improvements in muscle mass and function after the proposed muscle reinforcement program, whereas no significant differences were found in the POSTURAL group, after the adopted postural training. Furthermore, the muscle reinforcement program was able to induce in RESISTANCE group significant improvements in static balance parameters, whilst no significant differences in these values were found in the POSTURAL group, after the postural training. On the whole, the proposed muscle reinforcement program was able to induce positive effects both on sarcopenia and on postural parameters.

Sarcopenia is closely related to a risk of adverse outcomes, including poor quality of life and impaired ability to perform activities of daily living [8, 9], but, mostly important, it increases the risk of falls and fractures [10]. At the same time, the decrease in muscle strength could be also an important cause of postural instability [11], and, therefore, it represents an intrinsic risk factor for falling, by reducing the ability of older adults to adequately react to unexpected perturbations and to successfully regain balance [12–14].

Structured physical activity interventions are known to delay the onset of disability in older adults, improving clinical outcomes such as physical performance, gait speed, overall survival, fall risk, and quality of life [36]. So far, there are heterogeneous findings regarding the effects of exercise interventions on the maintenance of functional fitness, including sarcopenia [37].

Two milestone studies have been performed to standardize exercise interventions and elders related outcome measurements [38–40]. Namely, the LIFE study was aimed at investigating the effectiveness of physical interventions in sedentary community dwelling older adults without comorbidity. The main results indicated that the longitudinal improvement of functional fitness was a general positive end point by virtue of a structured moderate physical activity program compared with health education program. Although reduced major disability over 2.6 years was observed, high heterogeneity of important cut points was identified, representing a clinical important challenge.

The SPRINT study was also aimed at assessing longitudinal mobility disability prevention in older adults by virtue of combined nutritional and physical interventions to improve sarcopenia and physical frailty. This study was geared to produce systematic advancements in the management of frail older adults by a multifactorial set of interventions that included physical activity.

A recent systematic analysis has pointed out how exercise interventions are beneficial to body composition and muscle strength [41]. However, the training effect is generally inconsistent due to heterogeneity in exercise mode, duration, and intensity, which makes a generalization of training approaches impossible [42, 43].

To overcome this difficulty, we assigned a homogeneous group of seventy-two older women with moderate sarcopenia, according to EWGSOP criteria [8], to two types of 9-month adapted physical activity program: the first one focused on muscle reinforcement, and the second one focused on postural exercises. Due to the importance of a balanced diet for sarcopenic people [44], before starting the physical activity program all the participants were asked to answer to a self-administered food questionnaire that demonstrated that participants had a balanced daily intake of nutrients. We showed that the muscle reinforcement program significantly increased the absolute and the percent lean mass, the skeletal muscle mass (SM), and the skeletal muscle mass index (SMI), obtained by BIA predicted skeletal muscle mass (SM) equation (SM/height^2^). Furthermore, this type of APA program applied to the RESISTANCE group significantly improved handgrip strength (HGS), a parameter that well correlates with leg strength [8], sarcopenia, and physical function [43, 44]. The improvements in muscle mass, SMI, and muscle strength can be considered clinically relevant because the participants moved from a condition of moderate sarcopenia at baseline to a condition of normality after the muscle reinforcement intervention, according to the criteria used to diagnose sarcopenia.

It has been reported that postural control is improved by balance exercise intervention, whereas strength exercises or multicomponent interventions do not significantly influence such postural measurements [45]. In this study we investigated the effects of a muscle reinforcement protocol in improving balance parameters, such as Sway Path, Sway Area, Stay Time, and Spatial Distance, and we compared these effects with those obtained in another group of moderate sarcopenic older women that underwent an APA program focused on a postural training. Our findings showed that our muscle reinforcement program was more beneficial than the postural intervention in improving balance parameters of moderate sarcopenic older women. Therefore, under our experimental conditions, the results of this study suggest that the improvement of parameters for static balance can be more effectively obtained by the enhancement of muscle strength and functioning than by a postural training. This is an important achievement that obviously deserves further in-depth analysis, including the coassessment of other postural parameters, as well as the evaluation of potential biases.

A limitation of this study is the inability to establish a clear dose-response relationship along with individual functional fitness trajectories, due to the lack of baseline comprehensive geriatric assessment and polypharmacy analysis. In addition, the lack of osteoporosis assessment could prevent an accurate evaluation of osteosarcopenia, a common clinical condition in older people correlated with adverse clinical outcomes. On the other hand, the strengths of the study lie in the real-world assessment of an older adult population by a standardized adapted physical approach like that proposed in our APA project. Another limitation of the study is that we did not assess functional improvements in balance after the intervention period. It would be interesting to investigate, in further studies, whether the observed effects after a period of specific muscle reinforcement training are accompanied also by functional improvements.

## 5. Conclusions

The present findings show the effectiveness of a muscle reinforcement program on muscle mass and function, as well as on static balance parameters in moderate sarcopenic older women, thus suggesting that this type of intervention could represent a significant approach to reducing important risk factors for falling, such as sarcopenia and balance impairments. However, the limitations of this study and the potential biases must be considered before drawing firm conclusions, and further studies are required to deeply evaluate the effectiveness of different types of adapted physical activity program in muscle mass and function and in balance. On the whole, this study allows moving a step forward in the understanding of the clinical beneficial effects of adapted physical activity in an older population. The longitudinal assessment of this population, including physical activity training adherence over time, that is part of an ongoing study and the inclusion of geriatric assessment parameters will help understanding the elders risk stratification, on the basis of functional fitness and frailty prevention.

## Figures and Tables

**Table 1:** Participant characteristics at baseline. Data are means ± SD.

	RESISTANCE (n=35)	POSTURAL (n=37)	p-value
Age (years)	69.9±2.7	70.0±2.8	n.s.
Height (cm)	1.62±0.02	1.59±0.01	n.s.
Body mass (kg)	63.86±1.75	63.77±2.15	n.s.
Body mass index (kg/m^2^)	24.34±0.72	25.23±0.86	n.s.
Handgrip strength (kg) (mean of the two sides)	17.84±4.97	17.86±5.3	n.s.

**Table 2:** Static balance data expressed as median associated to the interquartile range. CE: closed eyes; OE: open eyes; n.s.: not significant.

	**RESISTANCE training group**			**POSTURAL training group**			**RESISTANCE vs. POSTURAL**
	**T0**	**T1**	**p**	**T0**	**T1**	**p**	
**Sway path (mm/s)**							
** CE**	16.78 [13.16,24.21]	15.44 [12.13, 20.59]	<0.001	19.57 [14.02, 24.96]	23.59 [15.82, 29.42]	<0.001	T0: n.s.; T1: p<0.01
** OE**	11.76 [9.27, 14.65]	9.75 [7.58, 11.76]	<0.001	12.13 [10.15, 13.68]	11.23 [10.16, 13.68]	n.s.	T0: n.s.; T1: p<0.05
**Sway area (mm** ^**2**^ **/s)**							
** CE**	40.64 [28.75, 59.39]	26.55 [16.85, 49.96]	<0.001	44.38 [20.04, 68.56]	52.25 [27.13, 76.98]	n.s.	T0: n.s.; T1: p<0.01
** OE**	19.36 [12.84, 32.15]	12.68 [9.14, 17.11]	<0.001	18.99 [11.58, 27.49]	17.74 [13.50, 24.89]	n.s.	T0: n.s.; T1: p<0.05
**Stay time (s)**							
** CE**	0.65 [0.49, 0.73]	0.87 [0.59, 1.10]	<0.001	0.59 [0.43, 0.88]	0.48 [0.35, 0.69]	<0.05	T0: n.s.; T1: p<0.001
** OE**	1.11 [0.81, 1.31]	1.37 [1.01, 1.85]	<0.001	0.99 [0.87, 1.38]	1.09 [0.89, 1.35]	n.s.	T0: n.s.; T1: p<0.01
**Spatial distance (mm)**							
** CE**	6.71 [4.51, 8.71]	4.65 [3.07, 6.97]	<0.001	6.19 [3.86, 9.41]	7.70 [4.79, 10.61]	<0.01	T0: n.s.; T1: p<0.01
** OE**	3.78 [2.55, 4.31]	2.61 [1.99, 3.41]	<0.001	3.28 [2.57, 4.33]	3.06 [2.74, 4.15]	n.s.	T0: n.s.; T1: p<0.05

## Data Availability

The data used to support the findings of this study are available from the corresponding author upon request.
